# Genome and transcriptome sequencing for inborn errors of immunity: a feasible multi-omics diagnostic approach

**DOI:** 10.3389/fimmu.2025.1510365

**Published:** 2025-03-28

**Authors:** Marija Rozevska, Katrina Daila Neiburga-Vigante, Inga Nartisa, Zane Lucane, Lota Ozola, Livija Bardina, Inta Jaunalksne, Natalija Gerula, Petra Krike, Gita Taurina, Ieva Nokalna-Spale, Ieva Micule, Baiba Vilne, Kai Kisand, Sander Pajusalu, Linda Gailite, Dmitrijs Rots, Natalja Kurjane

**Affiliations:** ^1^ Institute of Oncology and Molecular Genetics, Riga Stradiņš University, Riga, Latvia; ^2^ Children’s Clinical University Hospital, Riga, Latvia; ^3^ Riga Stradiņš University, Riga, Latvia; ^4^ University of Tartu, Tartu, Estonia; ^5^ Pauls Stradiņš Clinical University Hospital, Center of Clinical Immunology and Allergology, Riga, Latvia; ^6^ The Faculty of Medicine and Life Sciences, University of Latvia, Riga, Latvia; ^7^ Riga East University Hospital, Riga, Latvia; ^8^ Tartu University Hospital, Tartu, Estonia; ^9^ Department of Clinical Genetics, Erasmus Medical Centre, Rotterdam, Netherlands

**Keywords:** inborn errors of immunity, predominantly antibody deficiency, genome sequencing, transcriptome sequencing, variant of uncertain significance, complex structural variant, phenotypic correlation, diagnostic yield

## Abstract

Inborn errors of immunity (IEI), a diverse group of rare inborn disorders involving over 500 genes, pose diagnostic challenges despite next-generation sequencing advancements. Accurate molecular diagnosis is crucial for personalized treatment. This study aimed to assess the complementary role of genome and transcriptome sequencing in improving diagnostic yield for inborn errors of immunity. A cohort of 37 suspected IEI cases mainly consisting of predominantly primarily antibody deficiency (PAD) (27/37) underwent genome and transcriptome sequencing. We validated transcriptome sequencing analysis using positive controls and showed limitations of current methods. Among the 37 IEI cases, genetic etiology was identified in 14% (5/37). Genome and transcriptome sequencing prompted diagnostic changes in three initially diagnosed common variable immunodeficiency (CVID)/PAD cases, including showing RAS-associated autoimmune leukoproliferative disorder presenting as a novel CVID mimic disorder. The spectrum of identified pathogenic variants included *STAT1*, *ADA2*, *SH2D1A*, *NRAS*, and *NR2F1*. A complex structural variant in *SH2D1A* was characterized, demonstrating the significance of transcriptome sequencing in clarifying the genomic findings. While genome and transcriptome sequencing provided critical insights and allowed to provide correct diagnosis for at least 14% of the patients, the overall improvement in diagnostic yield over exome sequencing is limited. Transcriptome sequencing proved efficient in variant effect interpretation. Our findings underscore the evolving landscape of primary immunodeficiency genetics, necessitating ongoing exploration for novel genes and atypical phenotypes. The integration of genome and transcriptome sequencing holds promise but requires further refinement to enhance the diagnostic yield.

## Introduction

Inborn errors of immunity (IEI) represent a heterogeneous group of rare, inborn disorders, currently encompassing over 500 genes, with novel genes being discovered annually (1). Despite recent advancements and widespread application of the next-generation sequencing (NGS), the diagnostic yield for the majority of IEI groups remains low, except for severe combined immunodeficiencies (2, 3). The correct and precise IEI molecular diagnosis is crucial to providing personalized treatment and management (4).

Recently, the NGS advancements have allowed the application of genome sequencing (GS) in clinical diagnostics with the potential and hope to improve the diagnostic yield because of better gene coverage and detection of structural and non-coding variants (2). However, the interpretation of non-coding and/or structural variants remains a challenge, typically requiring additional functional analyses (3). To overcome some of these limitations, transcriptome sequencing (TS) can aid the functional interpretation of the variants identified using GS by providing insights into the qualitative (e.g., abnormal splicing) or quantitative (e.g., overexpression) RNA changes but is largely tissue-specific (5). Therefore, we argue that IEI represent a perfect group for inclusion of TS, as the affected tissues—immune cells—are easily available.

In this study, we aimed to improve the diagnostic yield by the complementary application of GS and TS in IEI. We have focused on adult individuals with predominantly antibody deficiencies (PAD), because historically this group has had a low diagnostic yield.

## Methods

This study recruited 37 individuals with inborn error of immunity (IEI), primarily focusing on a clinical diagnosis of common variable immunodeficiency (CVID) and predominantly antibody deficiency (PAD) (**Supplementary Tables E1, E2**). They represent all recognized PAD and CVID individuals in Latvia who consented for study and did not have prior genetic testing (6). Subjects were diagnosed with IEI using established European Society for Immunodeficiencies (ESID) criteria, including serum IgG and IgA and/or IgM deficiency with proven loss of antibody production. Additionally, for the TS validation and normalization, we have utilized 20 different previously diagnosed IEI cases with a confirmed monogenic cause, as well as 15 healthy individuals and 44 sIgA deficiency cases from previous studies (7, 8) (**Supplementary Table E1**).

All individuals and/or their legal guardians provided informed consent for this study. The study was approved by the Central Board of the Ethical Committee of the Health Ministry of the Republic of Latvia (No. 01-29.1/2878).

Peripheral blood was used to extract genomic DNA (EDTA tubes, BD, USA) and RNA (Tempus tubes, Thermo Fisher Scientific, Waltham, USA). Genome sequencing (150-bp paired-end read, at least 90 Gb) was performed using a PCR-free library and transcriptome sequencing (100-bp paired-end read, at least 80M clusters) was performed using KAPA RNA HyperPrep Kit with RiboErase Globin (Roche, Bazel, Switzerland), which includes rRNA and globin RNA removal. RNA integrity was assessed using RIN and DV200 values, with all samples passing quality control for transcriptome data analysis (RIN >6 and DV200 >97%). The sequencing was performed on Illumina NovaSeq 6000 (CEGAT, Tübingen, Germany).

Genome sequencing data were processed using standard pipeline including read mapping to the human genome (GRCh38) with BWA-mem (9) and short variant calling using DeepVariant (10). Structural variants were called Parliament2 (11)—a consensus structural variant caller utilizing multiple tools; short tandem repeats (STRs) were called using ExpansionHunter (12) using latest data from morbid STR database (STRipy) (13) and visualized with REViewer (14). Variant annotation and interpretation were performed using the seqr tool (15), focusing analysis on the known IEI-associated genes.

Transcriptome reads were mapped to human genome (GRCh38) using STAR (16), and further analysis was performed using the DROP package, analyzing expression outliers, splicing outliers, and monoallelic expression outliers, as well as the OutSingle tool focusing on the expression outlier analysis (p<0.05 and Z ≥ 3). The analysis was focused on the known IEI-associated genes. All analyzed samples (including healthy controls and validation samples) were processed by the DROP package and OutSingle tool in one batch to reduce expression variability among samples and account for common splicing events.

## Results

We have included and performed GS and TS on 37 cases (51% women) with suspected IEI, primarily with PAD (29/37) and symptom onset at adult age (23/37) ranging from 1 to 58 years, with a median of 29 years. RNA extraction failed for one of the individuals. Detailed clinical descriptions are provided in **Supplementary Table E1**.

We utilized the previously established DROP package (17), which identifies gene expression outliers (using OUTRIDER), splicing outliers (using FRASER), and monoallelic expression. As TS analysis requires larger cohorts, we additionally included TS data of 15 healthy individuals and 44 sIgA deficiency cases, as well as 20 different previously diagnosed IEI cases with a confirmed monogenic cause (**Supplementary Table E1**). The latter group was used to validate the TS pipeline: three cases had splicing variants, three cases—truncating variant predicted to undergo nonsense-mediated decay (NMD) in a gene expressed in blood, seven cases with a truncating variant in a gene not expressed in blood, and seven cases with a missense variant. While FRASER correctly identified all three splicing defects, OUTRIDER failed to detect most cases expected to result in a reduced expression and identified only a single case of reduced expression resulting from a splice variant (**Table 1**). To overcome this, we tested OutSingle (18), which performs less stringent normalization to provide higher sensitivity, as an alternative method. Indeed, OutSingle found two additional expression outliers among known variants (**Table 1**), while increasing the total number of detected outliers per case (from 1.5 to 85.5 on average). However, when limited to IEI genes, the difference was less prominent (from 0.1 to 2.2 outliers on average). Interestingly, OutSingle identified *ATM* variant NM_000051.4:c.7630-2A>C in a homozygous state, but not in a heterozygous state in a different sample (**Table 1**). This suggests that the TS data analysis is complex and requires additional time for result interpretation while also requiring improvements in established pipelines to increase sensitivity. Finally, two truncating variants of *ATM* gene NM_000051.4:c.5932G>T p.(Glu1978Ter) and *NBN* gene NM_002485.5:c.657_661del p.(Lys219AsnfsTer16)) were predicted to undergo NMD but were not identified as expression outliers by either of the methods and, upon visual inspection of the TS data, were identified to be exceptions from the known NMD rules (19) and escape NMD. As expected, missense variants and variants in genes with insufficient expression did not affect splicing, nor expression (data not shown).

**Table 1:** Blood transcriptome sequencing analysis results for the IEI cases with variants expected to affect RNA expression and/or splicing.

Nr.	Gene; genetic variant according to HGVS, genotype	Variant group	Expression outliers 1 (OUTRIDER; DROP)	Expression outliers 2 (OutSingle)	Splicing Outliers (FRASER; DROP)	MAE* (DROP)
Controls						
C43	*ATM;* NM_000051.4:c.7630-2A>C r.spl, heterozygous	Splice variant	Negative	Negative	pval = 3.01E-08; psi = 0.6; deltapsi = -0.4	NA
C44	*ATM;* NM_000051.4:c.7630-2A>C r.spl, homozygous	Splice variant	Negative	Z-score = -4.87; pval = 1.14E-06	pval = 1.66E-21; psi = 0.05; deltapsi = -0.94	NA
C55	*ATM;* NM_000051.4:c.5932G>T p.(Glu1978Ter) and c.8731A>C p.(Thr2911Pro), compound heterozygous	Nonsense (escape NMD) homozygous	Negative	Negative	Negative	NA
C53	*ADA2;* NM_001282225.2:c.464del p.(Pro155Hisfs*29) and c.506G>A p.(Arg169Gln), compound heterozygous	Nonsense	Negative	Z-score = -4.31; pval = 1.62E-05	Negative	NA
C16	*BTK;* NM_000061.3:c.391 + 1G>A, hemizygous	Splice variant	Z-score = -6.77; pval = 2.60E-05	Z-score = -7.60; pval = 2.96E-14	pval = 2.11E-34; psi = 0; deltapsi = -0.98	NA
C28	*NBN;* NM_002485.5:c.657_661del p.(Lys219AsnfsTer16), homozygous	Frameshift (escape NMD)	Negative	Negative	Negative	Negative
Patients						
P19	*SH2D1A* complex SV, hemizygous	Structural variant	Z-score = -7.29; pval = 2.64E-06	Z-score = -8.80; pval = 1.43E-18	Negative	Negative
P32	*NR2F1;* NM_005654.5:c.321_322del p.(Ser108Phefs*288), heterozygous	Frameshift (escape NMD)	Negative	Negative	Negative	Negative

HGVS, human genome variation society; SV, structural variation; NMD, nonsense-mediated decay; NA, not applicable; pval, *p* value.

*Raw genomic data were not available for the controls, so monoallelic expression was not analyzed.

**Figure 1:**
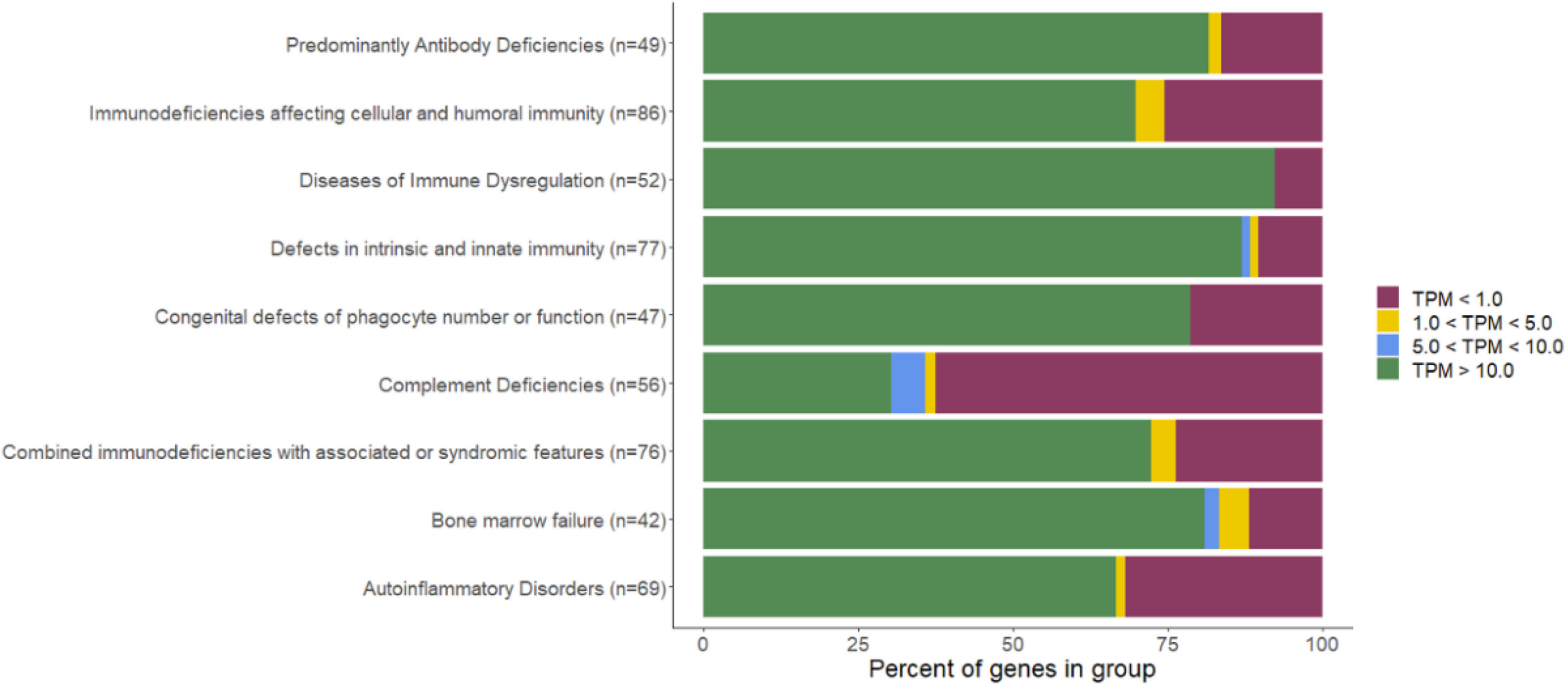
Percentage of genes with sufficient expression in blood per IEI group (IUIS 2022 classification). PM, transcripts per million.

Additionally, we evaluated the proportion of known IEI genes that have a sufficient expression in blood (based on the full TS cohort), to be analyzable. We divided the genes in groups based on the IUIS 2022 classification (20). While most of the PAD genes have a high expression in blood (TPM >10), it is variable between the groups, with less than half of the complement deficiency group genes having sufficient expression (**Figure 1**).

**Figure 2:**
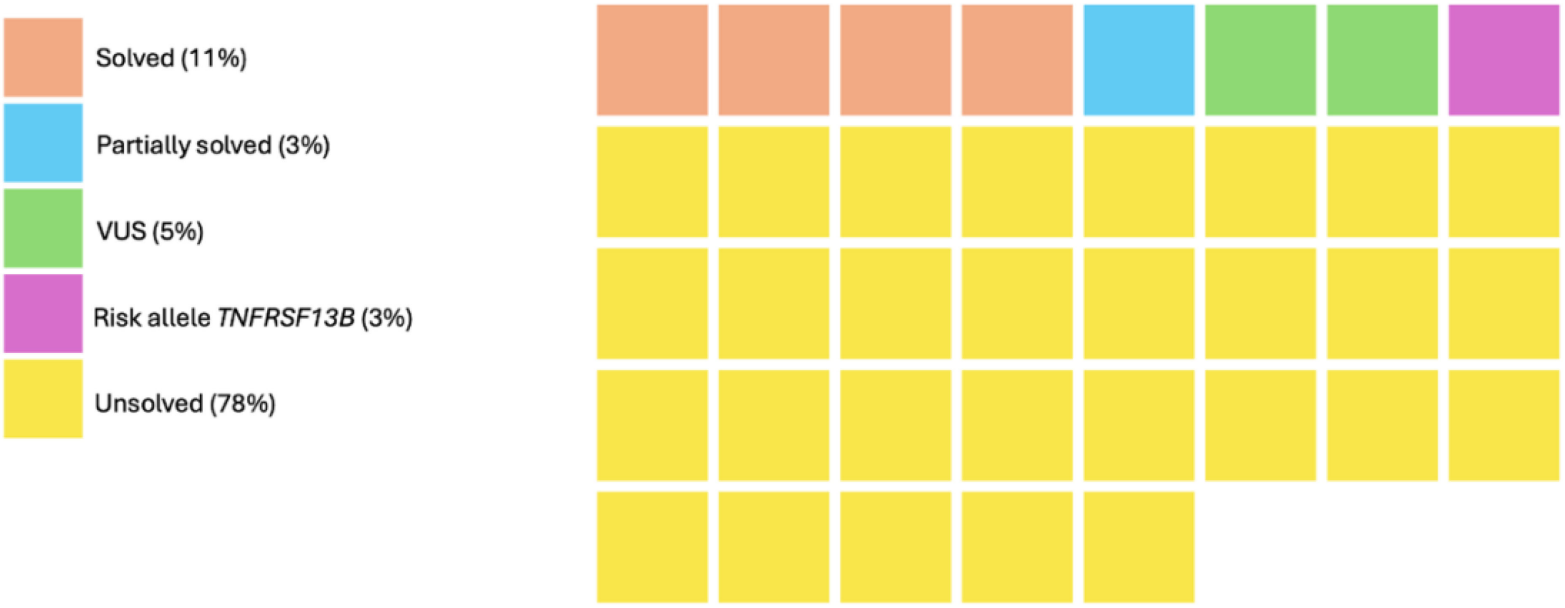
GS and TS testing outcomes in the cohort of 37 IEI. Each square represents a case. VUS, variant of uncertain significance.

Finally, we interpreted GS and TS data for 37 IEI cases (**Figure 2** and **Table 1**), focusing on the currently known 565 IEI-associated genes (online repository and **Supplementary Table E3**). We were able to identify genetic etiology in 5/37 (14%) patients (described below; **Supplementary Table E1**). Additionally, two cases had VUSs in the *NLRP1* and the *IKBKB* genes (**Supplementary Table E1**), but further functional tests and segregation analysis were not available. Finally, one patient (P31) with PAD was heterozygous for a known CVID risk factor *TNFRSF13B* NM_012452.2:c.542C>A p.(Ala181Glu).

Patient P6, a 32-year-old woman with suspected PAD who presented with reduced IgM and IgG4 and reduced numbers of CD4+ T cells with a medical history of atypical pneumonia (*M. bovis*), recurrent mild candida infections, vasculitis involving ascending aortic aneurysm, mild asthma, and transient presence of ANA autoantibodies, was initially treated with subcutaneous immunoglobulins and antifungals for *C. albicans* infection episodes. Genetic testing identified a pathogenic *STAT1* gain-of-function variant NM_007315.3:c.812A>C p.(Gln271Pro), which resulted in a change of diagnosis to 31C immunodeficiency (OMIM:614162), discontinuation of subcutaneous immunoglobulins, and subsequent vasculitis treatment with JAK-inhibitor.

Patient P19 is a 27-year-old man initially diagnosed with CVID at the age of 12, with recurrent viral and bacterial infections and hypogammaglobulinemia. Genetic testing unveiled a hemizygotic *SH2D1A* gene complex structural variant, affecting the transcription start site and exon 1. Initially, TS identified a reduced (but not absent) gene expression, and GS identified a single high-quality deletion, only partially covering the structural variant. Visual inspection, guided by these findings, uncovered a complex structural variant, subsequently confirmed by Sanger sequencing: NC_000023.11:g.124343810_124346883del;g.124347117_124348806del;g.124349109delins124346638_124346868. Despite the complexity of the structural variant, the patient did not exhibit typical X-linked lymphoproliferative disease (OMIM:300490) features, which might be explained by the residual gene expression, observed on TS. The patient is currently in remission under subcutaneous immunoglobulin treatment.

Patient P21, a 30-year-old woman with suspected PAD, had a history of recurrent infections, stomatitis, subfebrile temperature, and cytopenia, as well as reduced IgG2, IgM, and IgA levels. Genetic testing identified two (likely) pathogenic *ADA2* gene variants NM_001282225.2:c.506G>A p.(Arg169Gln) and c.620T>G p.(Phe207Cys) with the gnomAD (21) estimated probability of 100% that these variants occur in different haplotypes (i.e., in compound heterozygous state). The result likely confirms the diagnosis of *ADA2* deficiency (OMIM:615688).

Patient P10, a 27-year-old woman with a history of recurrent respiratory infections, autoimmunity, and hypogammaglobulinemia, was initially diagnosed with CVID at the age of 12 years old. Genetic testing revealed a pathogenic somatic *NRAS* variant NM_002524.5:c.35G>T p.(Gly12Val), with 30% allele frequency, resulting in a change of diagnosis to RAS-associated autoimmune leukoproliferative disorder (RALD) (OMIM:614470). The patient is currently in remission under subcutaneous immunoglobulin treatment.

Patient P32 is a 4-year-old man with epilepsy and developmental delay and had suspected PAD due to recurrent viral infections, pneumonia, otitis, and a specific antibody deficiency (no response after vaccination). Genetic testing revealed a pathogenic *NR2F1* gene variant NM_005654.5:c.321_322del p.(Ser108Phefs*288), confirming the diagnosis of Bosch–Boonstra–Schaaf Optic Atrophy Syndrome (BBSOAS) (OMIM:615772). Presently, no association is known between IEI and the BBSOAS (22) or *NR2F1* gene. Hence, the identified pathogenic variant currently only partially explains the phenotype.

## Discussion

In total, we have identified a genetic cause in at least 14% of cases, which is comparable with the other studies, ranging in diagnostic yield for PADs from 10% to 70% (2–4). No individual carried a pathogenic STR expansion (e.g., in *DMPK* gene causing myotonic dystrophy type 1, associated with hypogammaglobulinemia). Most of the identified variants in this study could also be identified using exome sequencing, so GS and TS combination currently failed to significantly improve the diagnostic yield. However, the *SH2D1A* structural variant would likely be missed without the GS and TS due to its complexity. Additionally, TS helped the interpretation of this variant by indicating the residual gene expression, which might explain the mild phenotype. Importantly, standard DROP pipeline failed to prioritize multiple positive controls with splicing defects, requiring use of the additional tool—OutSingle. Similarly, the Ensemble Parliament2 tool (11) failed to precisely define the complex SV in *SH2D1A* requiring additional Sanger sequencing. This highlights that improved tools are required for an improved GS and TS analysis. The diagnostic yield of GS and TS could be higher in the future with the discovery of novel genes, reinterpretation of VUSs, and improved non-coding variant analysis, as well as improved variant detection with novel tools. However, currently non-coding variants represent only a minority of the known IEI etiology, and we did not identify expression or splicing changes in genes that could explain patients’ phenotypes. This suggests that exome sequencing could remain the most cost-efficient option for routine IEI genetic diagnostics and can also provide additional diagnoses beyond coding short variant analysis with additional tools in a significant proportion of patients (23), whereas GS and TS can remain as a second-tier test for complex unsolved cases (24). While TS can reduce uncertainty for variants affecting splicing and gene expression and help classify such variants in the majority of IEI-associated genes (**Figure 1**), missense variants of uncertain significance still remain a challenge and would likely require further functional testing, which is currently rarely available in diagnostic settings.

Importantly, in all cases, an identified pathogenic variant resulted in a change of diagnosis, including three cases with initially diagnosed CVID/PAD. While *STAT1* is a well-known CVID mimic, *ADA2*- and *SH2D1A*-related disorders were only recently described as a cause of the CVID-like and CVID mimic disorders (25). Increasing application of genetic testing for IEI (including CVIDs and PADs) will likely identify novel genes and novel or atypical phenotypes for the known genes. Indeed, GS in 1,318 IEI demonstrated that ~25% of all diagnosed cases had a clinical presentation different from the one described (3). Similarly, we have also identified a somatic *NRAS* variant in an individual with an initial CVID diagnosis, adding the RALD into the CVID-like disorder spectrum. Furthermore, we cannot exclude that the BBSOAS (diagnosed in patient 6) could also be associated with immunodeficiency.

In conclusion, we show that genetic testing is crucial to providing correct diagnosis for IEI patients and has important clinical implications. While we were able to solve at least 13% of cases, the application of GS and TS currently provides limited improvement over exome (or targeted) sequencing. However, we show that TS can provide important insights for the GS-identified variant effect interpretation and might be efficient as a second-tier test.

## Data Availability

Clinical data are available on Dataverse DOI: 10.48510/FK2/4EMT9P.
